# Synthesis of new isoquinoline-base-oxadiazole derivatives as potent inhibitors of thymidine phosphorylase and molecular docking study

**DOI:** 10.1038/s41598-019-52100-0

**Published:** 2019-11-05

**Authors:** Khalid Zaman, Fazal Rahim, Muhammad Taha, Abdul Wadood, Syed Adnan Ali Shah, Qamar Uddin Ahmed, Zainul Amiruddin Zakaria

**Affiliations:** 1 Department of Chemistry, Hazara University, Mansehra, 21300 Khyber Pakhtunkhwa Pakistan; 2 Department of Clinical Pharmacy, Institute for Research and Medical Consultations (IRMC), Imam Abdulrahman Bin Faisal University, P. O. Box 1982, Dammam, 31441 Saudi Arabia; 3 Department of Biochemistry, Abdul Wali Khan University Mardan, Mardan, 23200 Pakistan; 4 Faculty of Pharmacy, Universiti Teknologi MARA Cawangan Selangor Kampus Puncak Alam, 42300 Bandar Puncak Alam, Selangor, D.E. Malaysia; 5 Atta-ur-Rahman Institute for Natural Products Discovery (AuRIns), Universiti Teknologi MARA Cawangan Selangor Kampus Puncak Alam, 42300 Bandar Puncak Alam, Selangor, D.E. Malaysia; 6 Department of Pharmaceutical Chemistry, Kulliyyah of Pharmacy, International Islamic University Malaysia, Pahang DM, Kuantan, 25200 Malaysia; 7 Department of Biomedical Science, Faculty of Medicine and Health Sciences, Universiti Putra Malaysia, 43400 Serdang, Selangor Malaysia; 8 Halal Institute Research Institute, Universiti Putra Malaysia, 43400 Serdang, Selangor Malaysia

**Keywords:** Enzymes, Computational chemistry

## Abstract

Here in this study regarding the over expression of TP, which causes some physical, mental and socio problems like psoriasis, chronic inflammatory disease, tumor angiogenesis and rheumatoid arthritis *etc*. By this consideration, the inhibition of this enzyme is vital to secure life from serious threats. In connection with this, we have synthesized twenty derivatives of isoquinoline bearing oxadiazole (1–20), characterized through different spectroscopic techniques such as HREI-MS, ^1^H- NMR and ^13^C-NMR and evaluated for thymidine phosphorylase inhibition. All analogues showed outstanding inhibitory potential ranging in between 1.10 ± 0.05 to 54.60 ± 1.50 *µ*M. 7-Deazaxanthine (IC_50_ = 38.68 ± 1.12 *µ*M) was used as a positive control. Through limited structure activity relationships study, it has been observed that the difference in inhibitory activities of screened analogs are mainly affected by different substitutions on phenyl ring. The effective binding interactions of the most active analogs were confirmed through docking study.

## Introduction

Cancer is considered as the second foremost cause of mortality world widely, therefore cancer therapy has gained considerable attention. Cancer therapy through target-based drug has developed many rationally designed inhibitors of thymidylate synthase^1–3^, glycinamide ribonucleotide formyltransferase^4^, and purine nucleoside phosphorylase^5^, that can impede the acute biochemical pathway or completely stop the DNA replication and subsequently prevent the growth of cancer cells. Ongoing research in this arena has identified TP as restorative target for cancer therapy. Thymidine phosphorylase is found in both prokaryotic and eukaryotic domains that accelerates the changeover of thymidine into thymine and 2′-deoxy-D-ribose 1-phosphates through reverse phosphorylsis^6–8^. Furthermore, dephosphorylation of 2′-deoxy-D-ribose 1-phosphates produces 2′-deoxy-D-ribose, which prompts the secretion of vascular endothelial growth factor (VEGF). Vascular endothelial growth factor favors a series of process like secretion of matrix metalloprotieneses, migration and proliferation of endothelial cells to tumor tissue, endorses cancer metastasis and instant generation of new blood vessels^9^. Moreover, high level of thymidine phosphorylase favors some cancerous problems such as breast tumor, colorectal, pancreatic, ovarian and hypoproiferative disease^10–13^.

Thymidine phosphorylase inhibitors suppress the growth of tumor cells via disintegrating the production of 2′-deoxy-D-ribose^14^. Currently, FDA has approved Lonsurf (trifluridine/tipiracil) as TP inhibiter, but it was found with some side effects like neutropenia, anemia and myelosuppression. Therefore, it is very crucial to develop TP inhibitors with least possible side effects and have the potentials to overcome the instant generation of new blood vessels and block the growth of tumor cells. In this regard, synthetic and medicinal chemists have reported various heterocyclic analogs as TP inhibitors^10,15–21^.

Isoquinoline alkaloids are *N*-containing heterocyle, widely distributed in nature and have manifested its broad spectrum potentials like immunoregulation, analgesic, anti-bacterial, anti-plateleted aggregation, anti-hypertensive and anti-arrhythmia^22,23^. Most of isoquinoline alkaloids have been confirmed for their therapeutic potentials against cancer related enzymes such as cyclin dependent kinase 4^24^, isosine 50-manophosphate dehydrogenase^25^, mammalian sterile 20 kinase^26^, topoisomerase1^27^ and 3-formylcoumarin.

**Figure 1:**
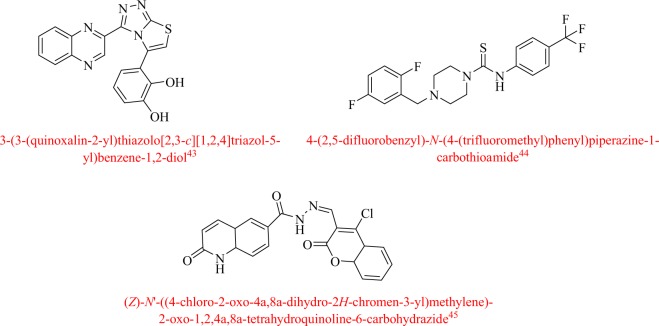
Reported lead candidates.

Our research group with continuous sincere efforts for many years have designed and synthesized various heterocyclic moieties with most promising potentials^28–41^. We have already reported quinoxaline, piperazine and 3-formylcoumarin analogues (Fig. 1) as potent TP inhibiter^42–44^.

**Figure 2:**
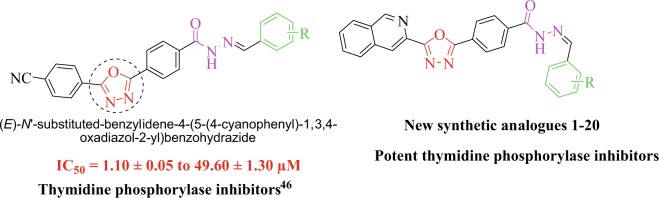
Rational study of the current work.

Comparatively with our previous work (Fig. 2)^45^, here, we have synthesized new isoquinoline based oxadiazole analogs for TP inhibitor to explore further their TP inhibitory potentials in search of lead candidate to limit the effect of TP over expression. The new compounds have been synthesized based on common features of our previously reported compounds.

## Results and Discussion

### Chemistry

**Figure 3:**
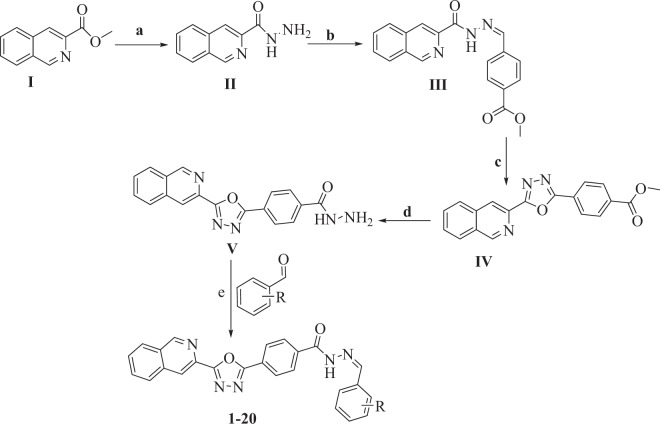
Reactions conditions: (**a**) N_2_H_4_.H_2_O, MeOH, Reflux, 6 h; (**b**) Methyl-4-formylbenzoate, MeOH, Reflux, 6 h; (**c**) PhI(OAc)_2_ DCM, rt; (**d**) N_2_H_4_.H_2_O, MeOH, Reflux, 6 h; (**e**) Substituted aromatic aldehydes/acetophenone, MeOH, 3–4 drops of AcOH, Reflux, 4 h.

Methyl isoquinoline-3-carboxylate (**I**) and hydrazine hydrate were reacted in methanol, refluxed for 6 hrs to give isoquinoline-3-carbohydrazide (**II**) as first intermediate product. The intermediate product (**II**) was further reacted with methyl-4-formyl benzoate in 20 ml ethanol, acidified the solution by few drops of acetic acid and refluxed the reaction mixture for 3 hrs to give methyl 4-((2-(isoquinoline-3-carbonyl) hydrazono)methyl)benzoate (**III**) as second intermediate product. The intermediate product (**III**) was then cyclized in DCM in the presence of phenyliodoacetate to give methyl 4-(5-(isoquinoline-3-yl)-1,3,4-oxadiazole-2-yl) benzoate (**IV**) as third intermediate product. The intermediate (**IV**) and hydrazine hydrate was mixed, then refluxed for six hrs in absolute methanol to give isoquinolinebenzohydrazide (**V**) as fourth intermediate product. Finally, the intermediate product (**V**) was treated with different benzaldehyde/acetophenone in 20 ml methanol in the presence of acetic acid and refluxed for 4 hrs to give isoquinoline bearing oxadiazole (**1**–**20**) as final product (Fig. 3, Table 1). The final product was washed with ethanol/hexane in order to obtain pure products. All analogues (**1-20**) of this compound library were elucidated by spectroscopic techniques like 1H-NMR, 13C-NMR and HREI-MS.

**Table 1:** Different notations for **R** on phenyl ring in isoquinoline bearing oxadiazole derivatives and their thymidine phosphorylase inhibitory activity. IC_50_ (Mean ± Standard Deviation).

S. No	R	IC_50_ value	S. No	R	IC_50_ Values
1		6.30 ± 0.20	11		5.30 ± 0.20
2		39.40 ± 0.90	12		4.60 ± 0.10
3		3.30 ± 0.10	13		6.00 ± 0.20
4		28.30 ± 0.50	14		27.40 ± 0.70
5		14.60 ± 0.40	15		47.30 ± 1.20
6		16.40 ± 0.50	16		18.50 ± 0.50
7		36.70 ± 0.90	17		22.10 ± 0.50
8		18.80 ± 0.50	18		7.30 ± 0.20
9		2.40 ± 0.10	19		54.60 ± 1.50
10		1.10 ± 0.10	20		1.10 ± 0.05
7-Deazaxanthine (7DX)			38.68 ± 1.12 *µ*M		

### *In vitro* thymidine phosphorylase activity

Twenty analogues (**1**–**20**) of isoquinoline bearing oxadiazole were synthesized and evaluated as inhibiter against thymidine phosphorylase enzyme. All analogs of the entire series were found with most potent inhibitory potential with IC_50_ values ranging in between 1.10 ± 0.05 *µ*M to 54.60 ± 1.50 *µ*M under positive control of reference drug 7-Deazaxanthine (IC_50_ = 38.68 ± 1.12 *µ*M). Seventeen analogues such as analogue **1–14** and **16–18** showed excellent inhibitory potential more better than the standard while three analogue **2**, **15** and **19** showed good inhibitory activity.

SAR study for thymidine phosphorylase inhibitory activity (**1–20**). The SAR study for particular analog among the series is mainly focused via the substitution on phenyl ring. Here in this study it was found that change of substituion or their swithing from one position to other postion on phenyl ring greatly effects the inhibitory appitude of analogues and these effects are summaraized in the below paragraphs.

If we compare analogue **15** (IC_50_ = 47.30 ± 1.20 *µ*M) having three methoxy groups at 2,3,4- position on phenyl ring with analogue **19** (IC_50_ = 54.60 ± 1.50 *µ*M) also having three methoxy groups on phenyl ring at 3,4,5-positions. Both analogues have the identical number of methoxy group on phenyl ring. The only difference found in their structures is the position of substituents on phenyl ring. With argue that substitution of methoxy groups on phenyl ring at 2,3,4-positions might activates the phenyl ring towards active of enzyme and therefore analogues **15** exhibited good activity profile than that of analogue **19** (Fig. S1).

It was seeming from the inhibitory activities that the position of substituents on phenyl ring greatly effects the inhibitory ability of the analogs. Therefore analogs **12**, **13** and **18** (IC_50_ = 4.60 ± 0.10 *µ*M, 6.00 ± 0.20 *µ*M and 7.30 ± 0.20 µM) all have the same hydroxyl and methoxy groups on phenyl ring at various positions but analog **12** have hydroxyl and methoxy groups at 3, 4 positions who exhibited superior inhibitory activity then analogs **13** and **18** respectively. It clearly indicates that the position of substituents on phenyl ring might play some basic role in binding interactions of ligands and active site of enzyme (Fig. S2).

It was also shown via SAR study that some time the inhibitory activity of analog was also increased by increasing the extent of identical substituents on phenyl ring. On this regard analog **3** (IC_50_ = 3.30 ± 0.10 *µ*M) with two chlorine atom at 2, 4 positions on phenyl ring displayed many fold better inhibitory activity then analog **6** (IC_50_ = 16.40 ± 0.50 *µ*M) bearing one more chlorine atom on phenyl ring at 4 position (Fig. S3).

Here in this study, it was clearly indicated that both electron donating group (EDG) and electron withdrawing group (EWG) on phenyl ring in all analog among the series displayed inhibitory activity. The slight difference in their potentials of analogs was mainly due to the position of substituent as well as the extent of substituent on phenyl ring. The binding interactions of the most active analogs and that of the active site of enzyme were confirmed through docking study.

### Docking study

**Figure 4:**
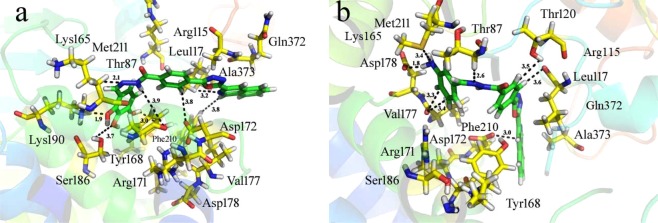
Docking conformations of compounds on thymidine phosphorylase enzyme. (**a**) 3D binding mode of compound **20** as inhibitor of thymidine phosphorylase enzyme. (**b**) 3D binding mode of compound **10** in binding cavity of thymidine phosphorylase enzyme. Ligands are shown green color.

**Figure 5:**
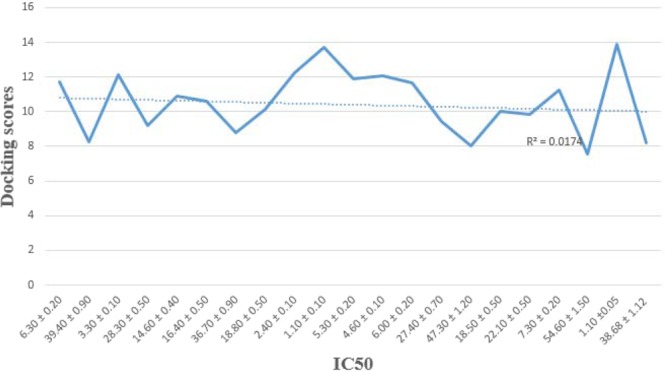
Predicted activity and IC_50_ correlation graph.

The concluded results of docking study of synthesized derivatives and that of thymidine phosphorylase enzyme assumed decent information about the binding pattern and have shown good correlation with experimental result. It was seeming with help of docking calculation that the top ranked conformation of all analogs approximately fitted in a good manner with active site of TP enzyme through various type of interactions *i*.*e* Thr 87, Arg 115, Leu 117, Met 211, Lys 165, Tyr 168, Arg 171, Ser 186, Asp 172, Val 177, Phe 210, Asp 178, Lys 190, Gln 372 and Ala 373 etc. The detail study about the binding interactions and docking score for almost all analogs are planned in the Table S1. The chemistry of substituents and the structural feature of most active analogs shown its efficiency in inhibitions like NH_2_ group have electron donating effect and certain electronegative groups *i*.*e* halogen, methoxy and OH groups. Among the docked analogs chlorine containing analogs were shown superior inhibition than Br supported by methoxy group. The interaction modes among the docked conformation of utmost analogs are demonstrated in Fig. 4(a,b). After docking, the docking conformation obtained was found with good docking score and show good result in-silico study, regarding thymidine phosphorylase enzyme inhibition. Rationally the biological evaluation and docking study of all analogs displayed good correlation result (Fig. 5).

## Conclusion

With variation through substituents on phenyl ring in the bulk molecule, twenty analogues (**1**–**20**) of isoquinoline bearing oxadiazole have been synthesized and screened for their thymidine phosphorylase activity. The screened result was found to be many folds better than standard drug 7-Deazaxanthine (IC_50_ = 38.68 ± 1.12 *µ*M). The structural elements in molecules were identified through docking study which play key role in the inhibitory activity. The activities of analogs were rationalized through structure-activity relationships (SAR) concluded that the whole series of analogs were comparatively identified as lead candidates which can serve for the development of new class of inhibitors.

## Experimental

### Material and methods

All chemicals and reagents of this protocol were purchased from sigma Aldrich, USA. All spectra’s ((^1^H-NMR, ^13^CNMR) were performed at 500 MHz. Initially all synthetic analogues of this protocol were confirmed through thin layer chromatography on TLC plate (Kieselgel 60, 254, E. Merck, Germany) and visualized by UV lamp 254 and 365 nm.

### General procedure for the synthesis of intermediate II

Methyl isoquinoline-3-carboxylate (I) was treated with hydrazine hydrate in methanol and refluxed for 6 hrs to afford isoquinoline-3-carbohydrazide (II) as first intermediate product. Isoquinoline-3-carbohydrazide (II) formation was checked through TLC. Furthermore, to increases its purity, the intermediates was recrystallized from methanol.

### General procedure for the synthesis of intermediate III

The intermediate product (II) was further reacted with methyl-4-formyl benzoate in absolute MeOH. The reaction mixture was acidified by 3–4 drops of glacial acid and then refluxed for 3 hrs to give methyl 4-((2-(isoquinoline-3-carbonyl)hydrazono)methyl)benzoate III as second intermediate product. The reaction mixture was stirred at room temperature till precipitate formation and filtered the crude mixture in order to obtain pure intermediate III.

### General procedure for the synthesis of intermediate IV

The intermediate product (III) was then cyclized in DCM at room temperature in the presence of phenyliodoacetate to give methyl 4-(5-(isoquinoline-3-yl)-1,3,4-oxadiazole-2-yl) benzoate IV as fourth intermediate product. Furthermore, the intermediate (IV) obtained was dissolved in 30 ml ethyl acetate and then added 50 ml distilled water and stirred for 35 minutes to make homogenous solution. The solution was separated layer wise in separating funnel in order to obtain pure intermediate IV.

### General procedure for the synthesis of intermediate V

The pure intermediate IV was than mixed with excess of hydrazine hydrate in MeOH and refluxed the reaction mixture for six hrs. The product formation was checked periodically through TLC. After completion the reaction, solvent was evaporated through rota-vapor to obtain pure intermediate V.

### General procedure for the synthesis of isoquinoline-base-oxadiazole analogues (1–20)

The pure intermediate (V) was reacted with various aromatic aldehydes/acetophenone in MeOH and acidified the solution by 3–4 drops of glacial acetic. The reaction mixture was refluxed for 4 hrs to obtain isoquinoline-base-oxadiazole compound library (1–20). In some cases, precipitate formation was taken place within the solution which was directly filtered to obtain pure analogues. Furthermore, in case of aldehydes/acetophenone having hydroxyl/methoxy substituents, the reaction mixture was dried openly in Petridish/china-dish and then followed by workup with hexane/methanol system to obtain pure analogues.

### 4-(5-(isoquinolin-3-yl)-1,3,4-oxadiazol-2-yl)-*N’*-(2-nitrobenzylidene)benzohydrazide (1)

Yield: 80%, ^1^H-NMR (500 MHz, DMSO- *d*_6_): δ 12.0 (s, 1H, NH), 9.5 (s, 1H, Ar- H), 8.9 (s, 1H, Aldehydic- H), 8.8 (s, 1H, Ar- H), 8.3 (m, 4H, Ar- H), 8.2 (d, *J* = 6.3 Hz, 1H, Ar-H), 8.1 (d, *J* = 6.4 Hz, 1H, Ar- H), 7.9 (d, *J* = 6.6 Hz, 1H, Ar- H), 7.8 (t, *J* = 6.1 Hz, 1H, Ar- H), 7.7 (m,3H, Ar- H), 7.6 (t, *J* = 6.4 Hz, 1H, Ar- H). ^13^CNMR (125 MHz, DMSO- *d*_6_): δ 164.3, 164.3, 163.1, 152.5, 150.1, 147.7, 143.1, 136.5, 134.8, 133.5, 132.6, 131.7, 130.2, 130.2 130.1, 130.0, 129.3, 128.0, 127.5, 127.4, 127.1, 126.6, 125.6, 124.0, 116.5. HREI-MS: *m/z* calcd for C_25_H_16_N_6_O_4_ [M]+ 464.1233, Found 464.1220.

### *N’*-(anthracen-9-ylmethylene)-4-(5-(isoquinolin-3-yl)-1,3,4-oxadiazol-2-yl)benzohydrazide (2)

Yield: 68%, ^1^H-NMR (500 MHz, DMSO- *d*_6_): δ 12.0 (s, 1H, NH), 9.5 (s, 1H, Ar- H), 8.9 (s, 1H, Aldehydic- H), 8.8 (s, 1H, Ar- H), 8.3 (m, 3H, Ar- H), 8.9 (q, 3H, Ar-H), 7.7 (m, 7H, Ar- H), 7.4 (m, 4H, Ar-H). ^13^CNMR (125 MHz, DMSO- *d*_6_): δ 164.3, 164.3 163.1, 152.5, 150.1, 143.2, 136.5, 132.5, 132.3 131.6, 131.7, 130.3, 130.1, 130.0, 129.2, 128.8, 128.6, 128.5, 128.3, 128.2, 128.0,127.9, 127.4, 127.2, 127.0, 126.7, 125.7, 125.6, 125.4, 125.3, 125.0, 123.6, 116.3. HREI-MS: *m/z* calcd for C_33_H_21_N_5_O_2_ [M]+ 519.1695, Found 519.1684.

### *N’*-(2,4-dichlorobenzylidene)-4-(5-(isoquinolin-3-yl)-1,3,4-oxadiazol-2-yl)benzohydrazide (3)

Yield: 78%, ^1^H-NMR (500 MHz, DMSO- *d*_6_) δ 11.9 (s, 1H, NH), 9.5 (s, 1H, Ar- H), 8.8 (s, 1H, Aldehydic- H), 8.7 (s, 1H, Ar- H) 8.3 (m, 3H, Ar- H) 8.2 (d, *J* = 6.6 Hz, 1H, Ar- H), 8.2 (m, 2H, Ar- H), 8.0 (d, *J* = 7.1 Hz, 1H, Ar- H), 7.9 (t, *J* = 6.3 Hz, 1H, Ar- H), 7.8 (t, *J* = 6.1 Hz, 1H, Ar- H), 7.7 (d, *J* = 6.6 Hz, 1H, Ar-H), 7.5 (d, *J* = 6.7 Hz, 1H, Ar- H). ^13^CNMR (125 MHz, DMSO- *d*_6_): δ 164.3, 164.3, 163.1, 152.2,150.1, 138.4, 136.5, 132.6,132.4, 131.0, 130.4, 130.2, 130.0, 129.3, 129.1, 129.0, 128.2, 128.0, 127.7, 127.4, 127.3, 127.0, 126.6, 125.5, 116.4. HREI-MS: *m/z* calcd for C_25_H_15_C_l2_N_5_O_2_ [M]+ 487.0603, Found 487.0590.

### 4-(5-(isoquinolin-3-yl)-1,3,4-oxadiazol-2-yl)-*N’*-(4-(phenoxymethyl)benzylidene)benzohydrazide (4)

Yield: 68%, ^1^H-NMR (500 MHz, DMSO- *d*_6_) δ 12 (s, 1H, NH), 9.5 (s, 1H, Ar- H), 8,8 (s, 1H, Aldehydic- H), 8.4 (s, 1H, Ar- H), 8.3 (m, 4H, Ar- H), 8.2 (d, J = 6.8 Hz, 1H, Ar- H), 8.1 (m, 2H, Ar- H), 7.9 (t, *J* = 6.2 Hz, 1H, Ar- H), 7.8 (t, *J* = 6.2 Hz, 1H, Ar- H), 7.7 (d, *J* = 6.1 Hz, 1H, Ar- H), 7.47 (m, 2H, Ar- H), 7.41 (m, 4H, Ar- H), 7.3 (t, *J* = 6.1 Hz, 1H, Ar- H), 4.9 (s, 2H, OCH2). ^13^CNMR (125 MHz, DMSO- *d*_6_): δ 164.3, 164.3, 163.1, 158.9, 152.1, 150.3, 146.6, 138.9, 136.5, 132.7, 132.2, 130.4, 130.3, 130.1, 129.9, 129.7, 129.5, 129.3, 129.2, 129.1, 128.9, 127.8, 127.8 127.4, 127.3, 127.0, 126.6, 125.4, 120.0, 116.6, 113.8, 113.6. HREI-MS: *m/z* calcd for C_32_H_23_N_5_O_3_ [M]+ 525.1801, Found 525.1787.

### 4-(5-(isoquinolin-3-yl)-1,3,4-oxadiazol-2-yl)-*N’*-(1-(4-nitrophenyl)ethylidene)benzohydrazide (5)

Yield: 68%, ^1^H-NMR (500 MHz, DMSO-d6) δ 11.9 (s, 1H, NH), 9.5 (s, 1H, Ar- H), 8.9 (s, 1H, Ar- H), 8.3 (m, 6H, Ar- H), 8.2 (m, 3H, Ar- H), 7.89 (t, *J* = 6 Hz, 1H, Ar- H), 7.87 (7.8 (t, *J* = 5.3 Hz, 1H, Ar- H), 7.7 (d, *J* = 6.2 Hz, 1H, Ar- H), 2.3 (s, 3H, CH3). ^13^CNMR (125 MHz, DMSO-d6): δ 164.3, 164.3, 163.1, 152.2, 150.1, 150.0, 147.5, 143.3, 136.4, 132.5, 130.6, 130.3, 130.1, 129.5, 128.3, 127.9, 127.7, 127.5, 127.3, 127.2, 127.1, 127.0, 126.7, 125.4, 116.5, 22.6. HREI-MS: *m/z* calcd for C_26_H_18_N_6_O_4_ [M]+ 478.1390, Found 478.1374.

### *N’*-(4-chlorobenzylidene)-4-(5-(isoquinolin-3-yl)-1,3,4-oxadiazol-2-yl)benzohydrazide (6)

Yield: 75%, ^1^H-NMR(500 MHz, DMSO- *d*_6_) δ 12.1(s, 1H, NH), 9.5 (s, 1H, Ar- H), 8,8 (s, 1H, Aldehydic- H), 8.7 (s, 1H, Ar- H), 8.3 (m, 4H, Ar- H), 8.2 (d, *J* = 6.7 Hz, 1 H,Ar- H), 7.9 (t, *J* = 6 Hz, 1H, Ar- H), 7.87 (m, 2H, Ar- H) 7.80 (m, 2H, Ar- H) 7.5 (m, 2H, Ar- H). ^13^CNMR (125 MHz, DMSO- *d*_6_): δ 164.3, 164.3, 163.1, 152.2, 150.1, 146.5, 136.5, 136.3, 132.5, 131.6, 130.6, 130.3, 130.1, 130.0, 129.8, 129.3, 128.7, 128.3, 128.0, 127.7, 127.3, 127.1, 126.5,125.4, 116.4. HREI-MS: *m/z* calcd for C_25_H1_6_ClN_5_O_2_ [M]+ 453.0993, Found 453.0977.

### *N’*-(5-bromo-2-methoxybenzylidene)-4-(5-(isoquinolin-3-yl)-1,3,4-oxadiazol-2-yl)benzohydrazide (7)

Yield: 75%, ^1^H-NMR(500 MHz, DMSO- *d*_6_) δ 12.1(s, 1H, NH), 9.5 (s, 1H, Ar-H), 8.8 (s, 1H, Aldehydic-H), 8.7 (s, 1H, Ar-H), 8.3 (m, 3H, Ar- H), 8.1 (m, 2H, Ar- H), 8.0 (d, *J* = 8.9 Hz, 1H, Ar- H), 7.9 (t, *J* = 7.7 Hz, 1H, Ar- H), 7.8 (t, *J* = 7.4 Hz, 1H, Ar- H), 7.7 (d, *J* = 6.4 Hz, 1H, Ar- H), 7.6 (d, *J* = 7.4 Hz, 1H, Ar- H), 7.1 (d, *J* = 7.4 Hz, 1H, Ar- H), 3.8 (s, 3H, OMe) ^13^CNMR (125 MHz, DMSO- *d*_6_): δ 164.3, 164.3, 163.1, 156.1, 152.2, 150.1, 146.2, 136.3, 134.5, 132.6, 131.3, 130.3, 130.0, 129.9, 129.5, 128.3, 127.3, 127.1, 127.0, 126.8, 125.5, 118.9, 116.6, 112.7, 110.2, 55.5. HREI-MS: *m/z* calcd for C_26_H_18_BrN_5_O_3_ [M]+ 527.0593, Found 527.0582.

### *N’*-(4-(dimethylamino)benzylidene)-4-(5-(isoquinolin-3-yl)-1,3,4-oxadiazol-2-yl)benzohydrazide (8)

Yield: 70%, ^1^H-NMR (500 MHz, DMSO-*d*_6_) δ 11.9 (s, 1H, NH), 9.5 (s, 1H, Ar-H), 8.8 (s, 1H, Aldehydic- H), 8.4 (s, 1H, Ar- H), 8.3 (m, 4H, Ar- H), 8.1 (d, *J* = 6.5 Hz, 1H, Ar- H), 7.9 (t, *J* = 5.9 Hz, 1H, Ar- H), 7.8 (t, *J* = 5.9 Hz, 1H, Ar- H), 7.6 (d, *J* = 6.9 Hz, 1H, Ar- H), 7.5 (d, *J* = 6.7 Hz, 2H, Ar- H), 6.7 (d, *J* = 7 Hz, 2H, Ar- H), 2.9 (s, 6H, N(CH3)2). ^13^CNMR (125 MHz, DMSO- *d*_6_): δ 164.3, 164.3, 163.1, 153.3, 152.2, 150.1, 146.5, 136.4, 132.5, 130.5, 130.3, 130.1, 129.3, 128.5, 128.3,128.0,127.4, 127.2, 127.1, 126.7, 125.7, 123.1, 116.6, 111.9, 111.8, 41.1, 41.0. HREI-MS: *m/z* calcd for C_27_H_22_N_6_O_2_ [M]+ 462.1804, Found 462.1789.

### *N’*-(3,5-dichloro-2-hydroxybenzylidene)-4-(5-(isoquinolin-3-yl)-1,3,4-oxadiazol-2-yl)benzohydrazide (9)

Yield: 55%,-^1^H-NMR (500 MHz, DMSO-*d*_6_) δ 11.9 (s, 1H, NH), 9.5 (s, 1H, Ar- H), 8.8 (s, 1H, Aldehydic- H), 8.6 (s, 1H, Ar-H), 8.3 (m, 2H, Ar- H), 8.2 (m, 3H, Ar- H), 7.9 (t, *J* =  = 6.2 Hz, 1H, Ar- H), 7.8 (t, *J* = 6.2 Hz, 1H, Ar- H), 7.7 (m, 2H, Ar- H), 7.6 (d, *J* = 1.9 Hz, 1H, Ar- H). ^13^CNMR (125 MHz, DMSO- *d*_6_): δ 164.3, 164.3, 163.1, 157.4, 152.2, 150.1, 146.3, 136.5, 133.9, 132.4, 130.1, 130.0, 129.9, 129.4, 128.4, 128.2, 128.0,127.7, 127.5, 127.2,126.6, 126.4, 125.4, 121.1, 116.5. HREI-MS: *m/z* calcd for C_25_H_15_Cl_2_N_5_O_3_ [M]+ 503.0552, Found 503.0536.

### *N’*-(3-aminobenzylidene)-4-(5-(isoquinolin-3-yl)-1,3,4-oxadiazol-2-yl)benzohydrazide (10)

Yield: 50%, ^1^H-NMR (500 MHz, DMSO-*d*_6_) δ 11.9 (s, 1H, NH), 9.5 (s, 1H, Ar- H), 8.8 (s, 1H, Aldehydic- H), 8.3 (s, 1H, Ar- H), 8.2 (m, 4H, Ar- H), 8.1 (d, *J* = 6.3 Hz, 1H, Ar- H), 7.9 (t, *J* = 5.8 Hz, 1H, Ar- H), 7.8 (t, J = 5.7 Hz, 1H, Ar- H), 7.6 (d, J = 6.8 Hz, 1H, Ar- H), 7.4 (d, J = 8.1 Hz, 1H, Ar- H), 7.2 (s, 1H, Ar- H), 7.1 (t, *J* = 6.7 Hz, 2H, Ar- H), 6.4 (s, 2H, NH2). ^13^CNMR (125 MHz, DMSO-*d*_6_): δ 164.3, 164.3, 163.1, 152.2, 150.1, 148.3, 146.4, 136.7, 134.3, 133.7, 132.6, 132.6 130.5, 130.3, 130.1, 129.3, 128.0, 127.5, 127.3, 126.5, 125.5, 119.1, 116.7, 117.4, 113.2. HREI-MS: *m/z* calcd for C_25_H_18_N_6_O_2_ [M]+ 434.1491, Found 434.1480.

### *N’*-(4-hydroxy-3,5-dimethoxybenzylidene)-4-(5-(isoquinolin-3-yl)-1,3,4-oxadiazol-2-yl)benzohydrazide (11)

Yield: 65%, ^1^H-NMR (500 MHz, DMSO-*d*_6_) δ 11.9 (s, 1H, NH), 9.5 (s, 1H, Ar- H), 8.8 (s, 1H, Aldehydic-H), 8.5 (s, 1H, Ar-H), 8.3 (m, 4H, Ar-H), 8.1 (d, *J* = 6.4 Hz, 1H, Ar- H), 7.9 (t, *J* = 6 Hz, 1H, Ar- H) 7.8 (t, *J* = 6 Hz, 1H, Ar-H), 7.6 (d, *J* = 6.6 Hz, 1H, Ar- H), 7.2 (d, *J* = 3.5 Hz, 2H, Ar- H), 3.8 (s, 6H, 2-OMe). ^13^CNMR (125 MHz, DMSO-*d*_6_): δ 164.3, 164.3, 163.1, 152.2, 150.1, 148.3, 148.1, 146.4, 139.5, 136.3, 132.7, 130.5, 130.3, 130.1, 129.4, 128.4, 128.1, 127.7, 127.5, 127.3, 126.4, 125.5, 116.3, 104.4, 104.1, 55.9, 55.9. HREI-MS: *m/z* calcd for C_27_H_21_N_5_O_5_ [M]+ 495.1543, Found 495.1528.

### *N’*-(3-hydroxy-4-methoxybenzylidene)-4-(5-(isoquinolin-3-yl)-1,3,4-oxadiazol-2-yl)benzohydrazide (12)

Yield: 78%, ^1^H-NMR (500 MHz, DMSO-*d*_6_) δ 11.9 (s, 1H, NH), 9.5 (s, 1H, Ar- H), 9.3 (s, 1H, Aldehydic-H), 8.8 (s, 1H, Ar- H), 8.3 (m, 6H, Ar- H), 8.2 (d, *J* = 6.6 Hz, 1H, Ar- H), 7.9 (t, *J* = 6.1 Hz, 1H, Ar- H), 7.8 (t, *J* = 6.1 Hz, 1H, Ar- H), 7.1 (d, *J* = 6.4 Hz, 1H, Ar- H), 7.1 (d, *J* = 6.7 Hz, 1H, Ar- H), 3.8 (s, 3H, OMe). ^13^CNMR (125 MHz, DMSO-*d*_6_): δ 164.3, 164.3, 163.1, 152.4, 152.2, 150.1, 147.1, 146.5, 136.5, 132.5, 130.9, 130.5, 130.3, 130.2, 129.3, 128.1, 127.8, 127.6, 127.4, 126.7, 125.6, 122.6, 116.5, 115.7, 112.1, 55.9. HREI-MS: *m/z* calcd for C_26_H_19_N_5_O_4_ [M]+ 465.1437, Found 465.1420.

### *N*’-(4-hydroxy-3-methoxybenzylidene)-4-(5-(isoquinolin-3-yl)-1,3,4-oxadiazol-2-yl)benzohydrazide (13)

Yield: 70%, ^1^H-NMR (500 MHz, DMSO-*d*_6_) δ 11.9 (s, 1H, NH), 9.5 (s, 1H, Ar- H), 8.8 (s, 1H, Aldehydic- H), 8.5 (s, 1H, Ar- H), 8.3 (m, 6H, Ar- H), 8.1 (d, *J* = 6.6 Hz, 1H, Ar- H), 7.9 (t, *J* = 6.1 Hz, 1H, Ar- H), 7.8 (t, *J* = 6.1 Hz, 1H, Ar- H), 7.1 (d, *J* = 6.4 Hz, 1H, Ar- H), 6.8 (d, *J* = 6.4 Hz, 1H, Ar- H), 3.8 (s, 3H, OMe). ^13^CNMR (125 MHz, DMSO-*d*_6_): δ 164.3, 164.4, 163.1, 152.5, 151.1, 149.2, 146.9, 136.5, 132.6, 132.6, 130.7, 130.5, 130.5, 129.9, 129.4, 128.2, 127.5, 127.3, 127.2, 126.5, 125.6, 122.7, 117.1, 116.4, 112.2, 55.9. HREI-MS: *m/z* calcd for C_26_H_19_N_5_O_4_ [M]+ 465.1437, Found 465.1420.

### *N*’-(3-cyanobenzylidene)-4-(5-(isoquinolin-3-yl)-1,3,4-oxadiazol-2-yl)benzohydrazide (14)

Yield: 65%, ^1^H-NMR (500 MHz, DMSO-*d*_6_) δ 11.9 (s, 1H, NH), 9.5 (s, 1H, Ar- H), 8,8 (s, 1H, Aldehydic- H), 8.5 (s, 1H, Ar- H), 8.3 (m, 4H, Ar- H), 8.2 (m, 4H, Ar- H), 8.1 (d, *J* = 6.4 Hz, 1H, Ar- H), 7.9 (t, *J* = 6.3 Hz, 1H, Ar- H), 7.8 (t, *J* = 6.3 Hz, 1H, Ar- H) 7.1 (d, *J* = 6.2 Hz, 1H, Ar- H). ^13^CNMR (125 MHz, DMSO-*d*_6_): δ 164.3, 164.3, 163.1, 152.2, 150.1, 146.8, 136.7, 134.5, 134.1, 133.3, 132.7, 132.2, 130.5, 130.2, 130.1,129.7, 129.5, 128.0, 127.6, 127.3, 127.1, 126.6, 125.6, 118.3, 116.5, 112.4. HREI-MS: *m/z* calcd for C_26_H_16_N_6_O_2_ [M]+ 444.1335, Found 444.1322.

### 4-(5-(isoquinolin-3-yl)-1,3,4-oxadiazol-2-yl)-*N*’-(3,4,5-trimethoxybenzylidene)benzohydrazide (15)

Yield: 55%, ^1^H-NMR (500 MHz, DMSO-*d*_6_) δ 12.09 (s, 1H, NH), 9.5 (s, 1H, Ar- H), 9.3 (s, 1H, Aldehydic- H), 8.8 (s, 1H, Ar- H), 8.5 (m, 4 H, Ar- H), 8.2 (d, *J* = 6.7 Hz, 1H, Ar- H), 8.1 (m, 2H, Ar- H), 7.9 (t, *J* = 6.1 Hz, 1H, Ar- H) 7.8 (t, *J* = 6.1 Hz, 1H, Ar- H), 7.2 (d, *J* = 6.5 Hz, 1H, Ar- H), 3.8 (s, 9H, 3-OMe) ^13^CNMR (125 MHz, DMSO-*d*_6_): δ 164.3, 164.3, 163.1, 153.4, 153.2, 152.2, 150.1, 146.5, 141.3, 136.4, 132.5, 130.3, 130.1, 130.0, 129.3, 128.3, 128.1, 127.6, 127.4, 127.2, 126.8, 125.4, 116.3, 104.0, 104.0, 60.5, 55.9, 55.9. HREI-MS: *m/z* calcd for C_28_H_23_N_5_O_5_ [M]+ 509.1699, Found 509.1685.

### *N’*-(2-cyanobenzylidene)-4-(5-(isoquinolin-3-yl)-1,3,4-oxadiazol-2-yl)benzohydrazide (16)

Yield: 68%, ^1^H-NMR (500 MHz, DMSO-*d*_6_) δ 12.4 (s, 1H, NH), 9.5 (s, 1H, Ar- H), 9.3 (s, 1H, Aldehydic- H), 8.9 (s, 1H, Ar- H), 8.3(m, 4H, Ar- H), 8.2 (m, 3H, Ar- H), 8.1 (d, (d, *J* = 6.4 Hz, 1H, Ar- H),7.10 (m, 1H, Ar- H), 7.9 (t, *J* = 6.3 Hz, 1H, Ar- H), 7.8 (t, *J* = 6.1 Hz, 1H, Ar- H), 7.6 (d, *J* = 5.3 Hz, 1H, Ar- H). ^13^CNMR (125 MHz, DMSO-*d*_6_): δ 164.3, 164.3, 163.1, 152.2, 150.1, 143.1, 136.5, 134.3, 132.6, 132.2, 131.5, 133.1, 130.1, 130.0, 129.9, 129.6, 129.3, 128.2, 127.5, 127.3, 127.2, 126.7, 125.7, 116.6, 115.6, 111.7. HREI-MS: *m/z* calcd for C_26_H_16_N_6_O_2_ [M]+ 444.1335, Found 444.1322.

### *N’*-(4-cyanobenzylidene)-4-(5-(isoquinolin-3-yl)-1,3,4-oxadiazol-2-yl)benzohydrazide (17)

Yield: 73%, ^1^H-NMR (500 MHz, DMSO-*d*_6_) δ 12.3 (s, 1H, NH), 9.5 (s, 1H, Ar- H), 8.8 (s, 1H, Aldehydic- H), 8.7 (s, 1H, Ar- H), 8.3 (m, 4H, Ar-H) 8.2 (m, 4H, Ar-H), 8.1 (d, *J* = 6.7 Hz, 1H, Ar- H), 7.9 (t, *J* = 6.2 Hz, 1H, Ar- H), 7.8 (t, *J* = 6.8 Hz, 1H, Ar- H), 7.6 (d, *J* = 6.8 Hz, 1H, Ar-H). ^13^CNMR (125 MHz, DMSO-*d*_6_): δ 164.3, 164.3, 163.1, 152.2, 150.1, 146.5, 138.0, 136.5, 132.4, 132.1, 132.0, 129.5, 130.5, 130.2, 130.0, 128.0, 127.7, 127.5, 127.3, 126.9, 126.3, 126.1, 125.6, 118.4, 116.5, 114.5. HREI-MS: *m/z* calcd for C_26_H_16_N_6_O_2_ [M]+ 444.1335, Found 444.1322.

### *N’*-(2-hydroxy-3-methoxybenzylidene)-4-(5-(isoquinolin-3-yl)-1,3,4-oxadiazol-2-yl)benzohydrazide (18)

Yield: 73%, ^1^H-NMR (500 MHz, DMSO-*d*_6_): δ 12.3 (s, 1H, NH), 9.5 (s, 1H, Ar- H), 8.8 (s, 1H, Aldehydic- H), 8.7 (s, 1H, Ar- H), 8.3 (m, 4H, Ar- H), 8.2 (d, *J* = 6.4 Hz, 1H, Ar- H), 7.9 (t, *J* = 6.1 Hz, 1H, Ar- H), 7.8 (t, *J* = 6 Hz, 1H, Ar- H), 7.2 (d, *J* = 6.2 Hz, 1H, Ar- H), 7.06 (d, *J* = 6.2 Hz, 1H, Ar- H), 6.9 (m, 2H, Ar- H), 3.8 (s, 3H, OMe). ^13^CNMR (125 MHz, DMSO-*d*_6_): δ 164.3, 164.3, 163.1, 152.2, 150.1, 149.9, 149.3, 146.0, 136.6, 132.7, 130.4, 130.4 130.1, 130.0, 129.3, 128.1, 127.7, 127.1, 126.6, 125.5, 124.2, 119.3, 116.5, 116.4, 115.0, 55.9. HREI-MS: *m/z* calcd for C_26_H_19_N_5_O_4_ [M]+ 465.1437, Found 465.1420.

### 4-(5-(isoquinolin-3-yl)-1,3,4-oxadiazol-2-yl)-*N*’-(2,3,4-trimethoxybenzylidene)benzohydrazide (19)

Yield: 60%, ^1^H-NMR (500 MHz, DMSO-*d*_6_) δ 12.0 (s, 1H, NH) 9.5 (s, 1H, Ar- H) 8.8 (s, 1H, Aldehydic- H), 8.7 (s, 1H, Ar-H), 8.3 (m, 5H, Ar- H), 8.2 (d, *J* = 6.6 Hz, 1H, Ar- H), 7.9 (t, *J* = 6.2 Hz, 1H, Ar- H) 7.8 (t, *J* = 6.2 Hz, 1H, Ar- H), 7.6 (d, *J* = 7.2 Hz, 1H, Ar- H) 6.9 (d, *J* = 7.4 Hz, 1H, Ar- H) 3.8 (s, 9H, 3-OMe). ^13^CNMR (125 MHz, DMSO-*d*_6_): δ 164.3, 164.3 163.1, 158.2, 151.3, 152.2, 150.1, 146.2, 143.3, 136.6, 132.7, 132.7, 130.1, 130.1 129.2, 128.3, 127.5, 127.3, 127.1, 126.7, 125.5, 125.3, 116.3, 110.1, 103.9, 61.3, 60.5, 55.9. HREI-MS: *m/z* calcd for C_28_H_23_N_5_O_5_ [M]+ 509.1699, Found 509.1685.

### 4-(5-(isoquinolin-3-yl)-1,3,4-oxadiazol-2-yl)-*N*’-(2,3,4-trihydroxybenzylidene)benzohydrazide (20)

Yield: 54%, ^1^H-NMR (500 MHz, DMSO-*d*_6_) δ 12.2 (s, 1H, NH) 9.5 (s, 1H, Ar-H) 8.8 (s, 1H, Aldehydic- H), 8.7 (s, 1H, Ar- H), 8.3 (m, 4H, Ar- H), 8.2 (d, *J* = 6.6 Hz, 1H, Ar- H), 7.9 (t, *J* = 6.1 Hz, 1H, Ar- H), 7.8 (t, *J* = 6.1 Hz, 1H, Ar-H), 7.6 (d, *J* = 7.4 Hz, 1H, Ar- H), 6.8 (d, *J* = 6.9 Hz, 1H, Ar- H), 8.4 (d, *J* = 6.9 Hz, 1H, Ar- H). ^13^CNMR (125 MHz, DMSO-*d*_6_): δ 164.3, 164.3, 163.1, 153.2, 152.5, 152.2, 150.1, 146.3, 136.5, 136.3, 132.6, 130.4, 130.3, 130.0, 129.2, 128.2, 127.4, 127.2, 127.1, 126.7, 126.4, 125.7, 116.5, 112.3, 110.2. HREI-MS: *m/z* calcd for C_25_H_17_N_5_O_5_ [M]+ 467.1230, Found 467.1213.

### Assay protocol for thyimidine phosphorylase

The desired activity of thymidine phosphorylase under this protocol was carried out with slight modification in the method described by Krenitsky and Bush in 1979^46,47^. Here, TP activity was determined through spectrophotometrically by absorbance at 290 nm. Although the whole reaction mixture of 200 µL have the limit of 145 *µ*L of potassium phosphate buffer (pH 7.4, 50 mM), 30 *µ*L of enzyme (human and *E. coli*) by concentration 0.05 and 0.002 U, distinectly, followed by incubation through 5 *µ*L of test materials (0.075, 0.15, 0.3 and 0.5 mM) at 25 °C for 10 min in triplet reader. Subsequently, pre-read at 290 nm was taken to conclude the absorbance of concern substrate particles. The crave substrate “thyimidin” (20 *µ*L, 1.5 mM), was then dissolved in the buffer (potassium phosphate), followed by instant addition to plate with constant read after every 10, 20 and 30 min in micro plate reader (spectra max, molecular devices, CA, USA). All the assay were implemented in triplicate under positive control of 7-Deazxanthine.

### Molecular docking

The interactions between target and inhibitors were investigated through molecular docking^48^. Molecular docking was carried out through MOE-Dock program (www.chemcomp.com) to find the interactions between synthesized analogs and ligand proteins. From Protein Databank (PDB), three dimensional structures (3D) of thymidine phosphorylase (4EAD) was retrieved. In MOE (www.chemcomp.com) the synthesized analogs were docked into the active site of the target docked enzyme by applying the default parameter i-e Rescoring 1: London dG, Refinement: Forcefield (MMFF94x), Placement: Triangle Matcher, London dG: Rescoring 2. Ten conformations were generated for each in which the conformation of the top ranked based on docking score was selected for additional studies in molecular docking. The best pose here having polar, Pi-H and H-Pi interactions were analyzed by Pymol software after molecular docking.

## Supplementary information

supporting data
