# Supplementary material for: Synthesis of new isoquinoline-base-oxadiazole derivatives as potent inhibitors of thymidine phosphorylase and molecular docking study
Source: Sci Rep. 2019 Nov 5;9:16015. doi: 10.1038/s41598-019-52100-0 (PMC6831664; doi:10.1038/s41598-019-52100-0)
Supplement: Supplementary file 1 — supporting data [file 41598_2019_52100_MOESM1_ESM.pdf]

Supplementary Data

Synthesis of new isoquinoline-base-oxadiazole derivatives as potent inhibitors of thymidine phosphorylase and molecular docking study

Khalid Zaman[a], Fazal Rahim[a], Muhammad Taha*[b], Abdul Wadood[c], Syed Adnan Ali Shah[d,e], Qamar Uddin Ahmed[f], Zainul Amiruddin Zakaria*[g,h]

[a]Depatment of Chemistry, Hazara University, Mansehra-21300, Khyber Pakhtunkhwa, Pakistan
[b]Department of clinical pharmacy, institute for research and medical consultations (IRMC), Imam Abdulrahman Bin Faisal University, P. O. Box 1982, Dammam 31441, Saudi Arabia.
[c]Department of Biochemistry, Abdul Wali Khan University Mardan, Mardan-23200, Pakistan.
[d]Faculty of Pharmacy, Universiti Teknologi MARA Puncak Alam Campus, 42300 Bandar Puncak Alam, Selangor D.E., Malaysia.
[e]Atta-ur-Rahman Institute for Natural Products Discovery (AuRIns), Universiti Teknologi MARA Puncak Alam Campus, 42300 Bandar Puncak Alam, Selangor D.E., Malaysia.
[f]Department of Pharmaceutical Chemistry, Kulliyyah of Pharmacy, International Islamic University Malaysia, Pahang DM, Kuantan 25200, Malaysia.
[g]Department of Biomedical Science, Faculty of Medicine and Health Sciences, Universiti Putra Malaysia, 43400 Serdang, Selangor, Malaysia
[h]Halal Institute Research Institute, Universiti Putra Malaysia, 43400 Serdang, Selangor, Malaysia

Table of Contents Page #

1. Compound # 1 Proton NMR 2
2. Compound # 7 Proton NMR 3
3. Compound # 13 Proton NMR 4
4. Compound # 18 Proton NMR 5
5. Compound # 18 Carbon NMR 6
6. Compound # 19 Proton NMR 7
7. Compound # 19 Carbon NMR 8
8. Figure S1 9
9. Figure S2 10
10. Figure S3 11
11. Table S1 12

Supplementary Data

Compound NO-1

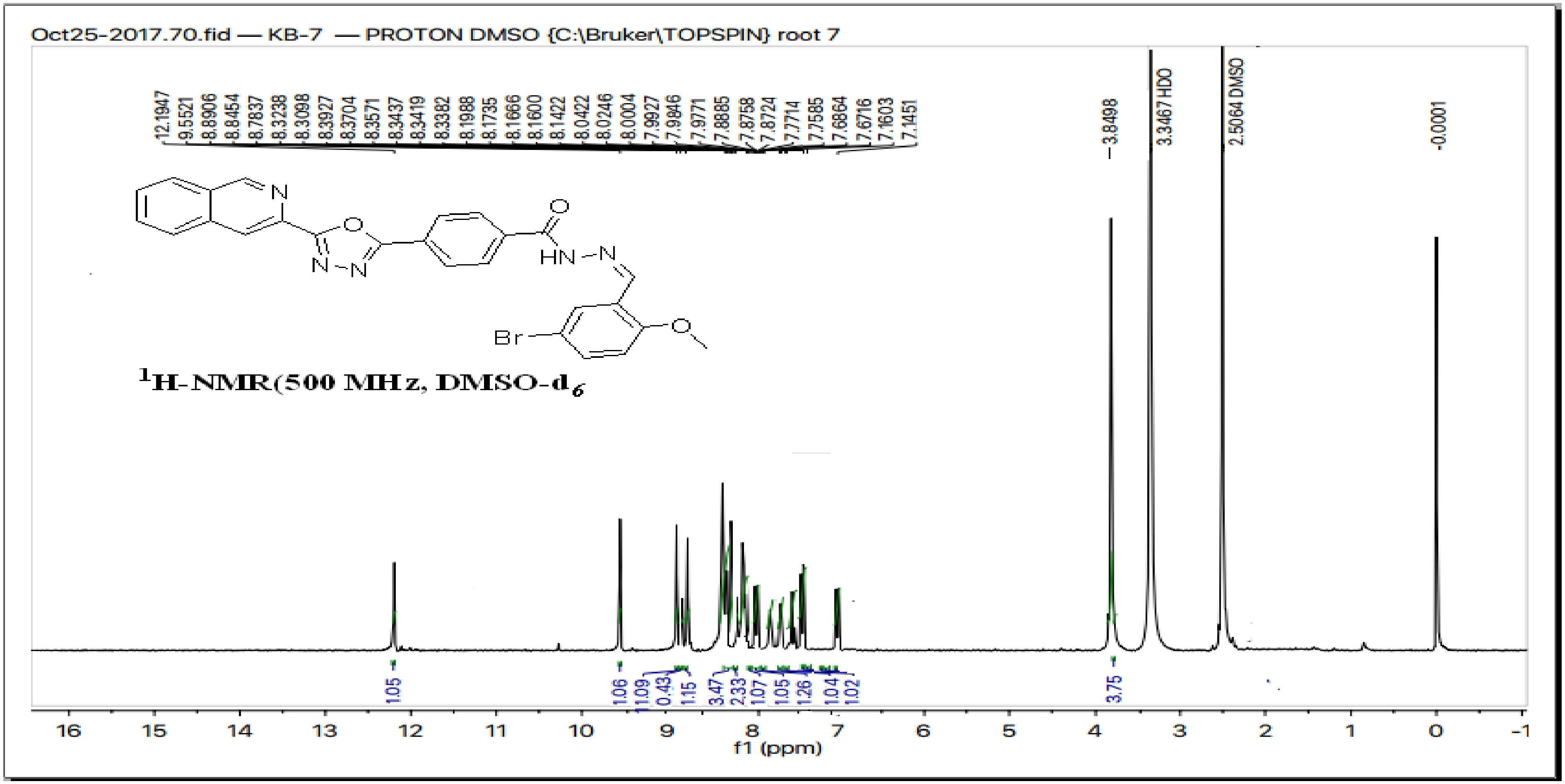

Supplementary Data

Compound NO-7

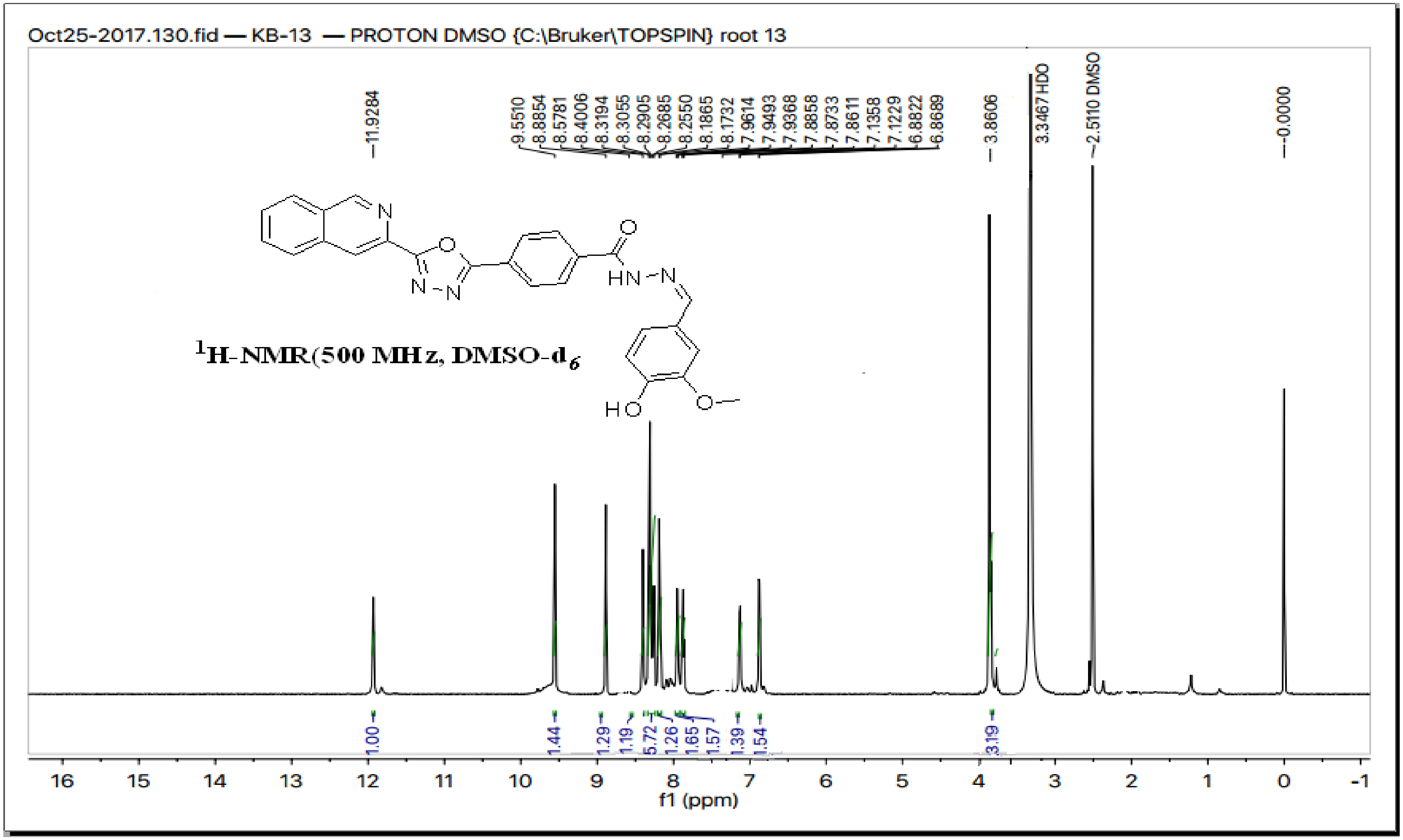

Supplementary Data

Compound NO-13

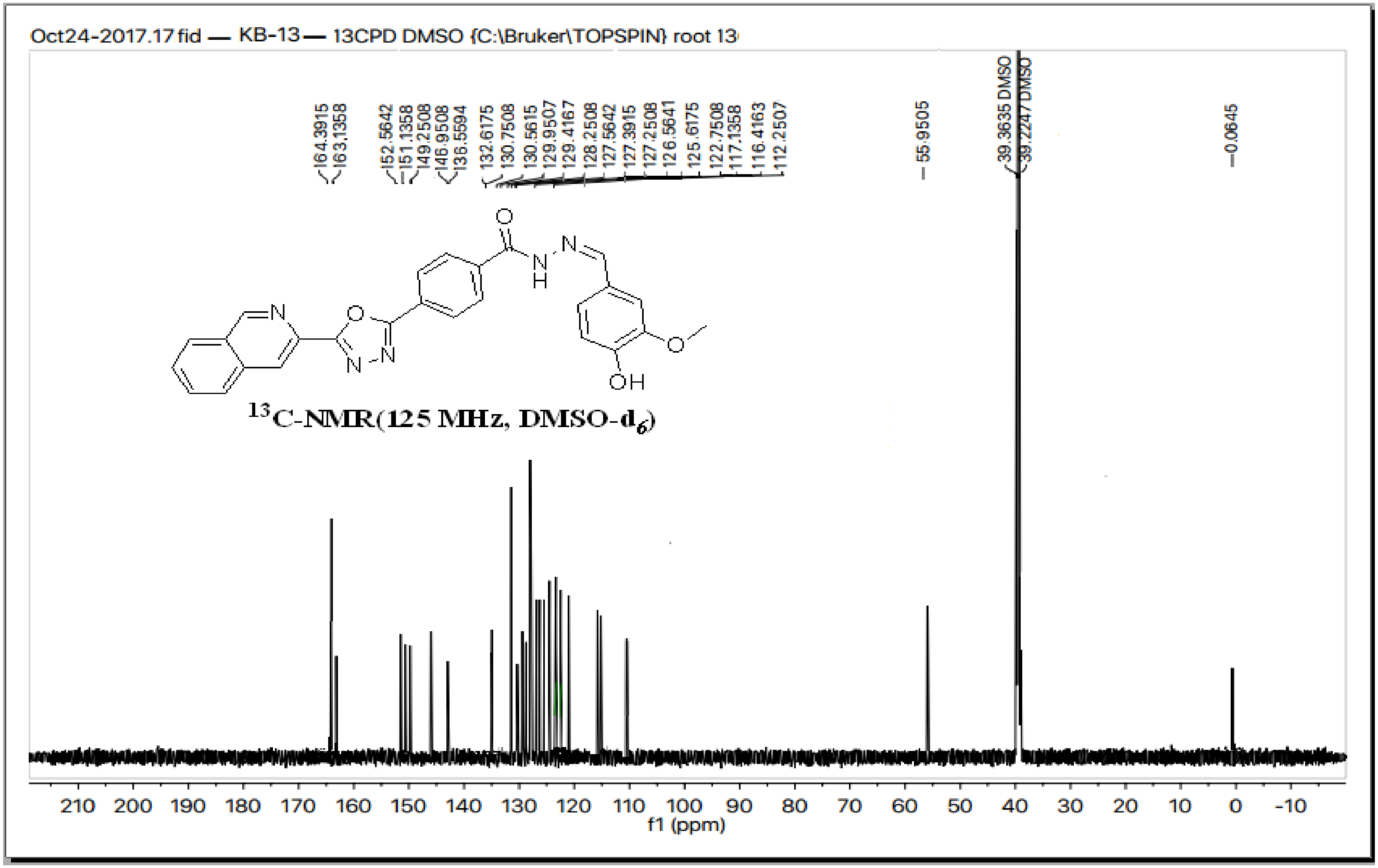

Supplementary Data

Compound NO-18

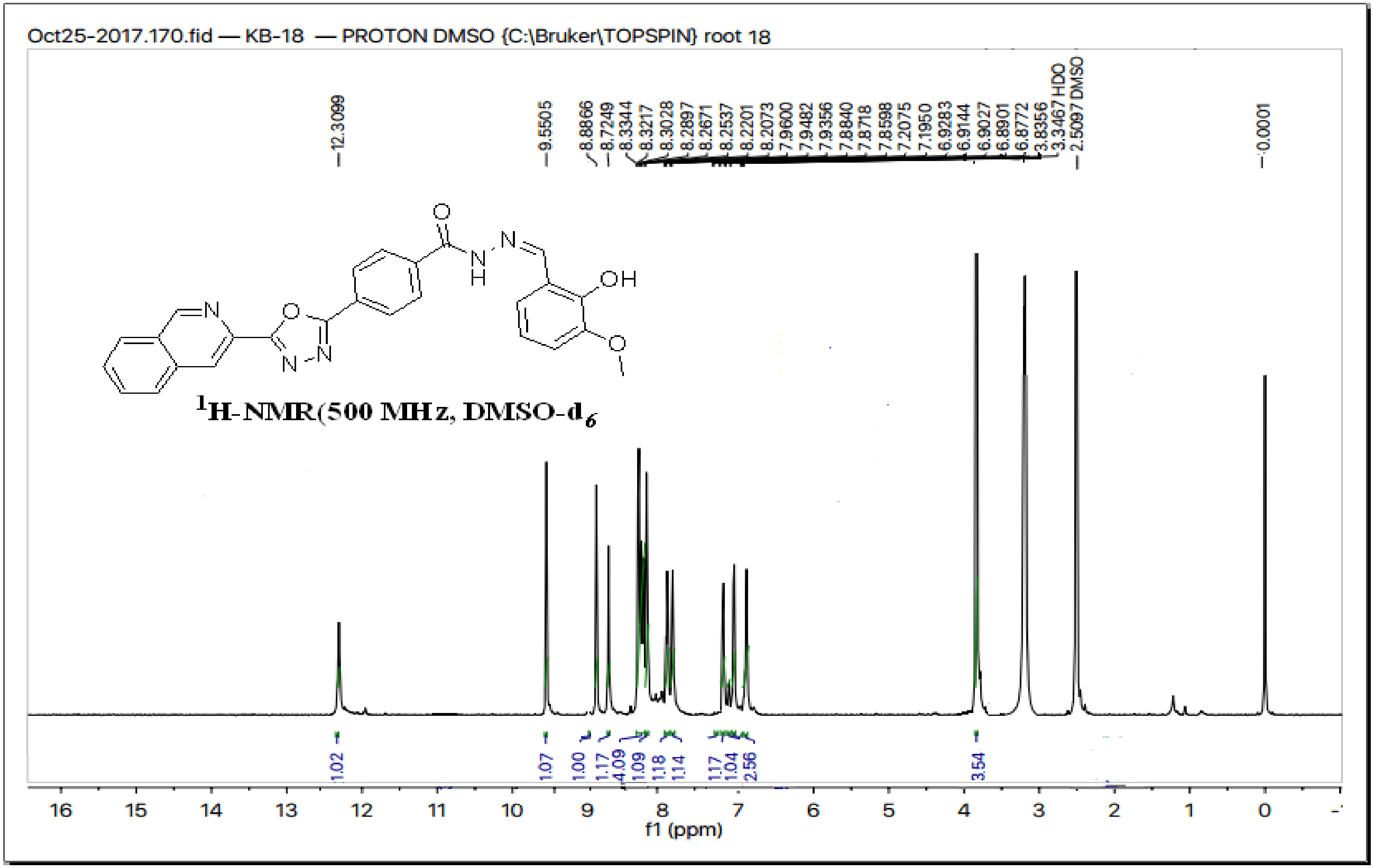

Supplementary Data

Compound NO-18

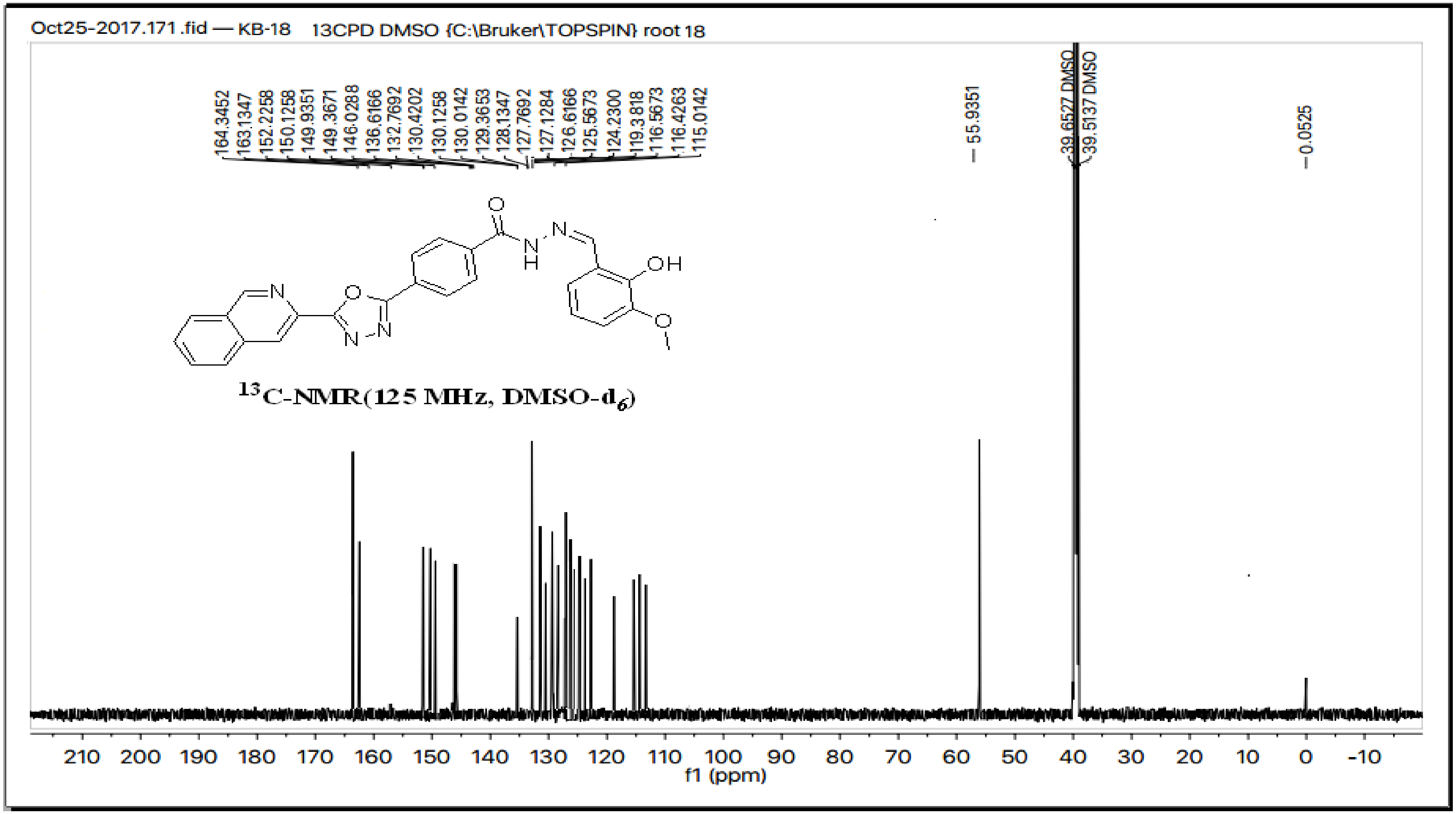

Supplementary Data

Compound NO-19

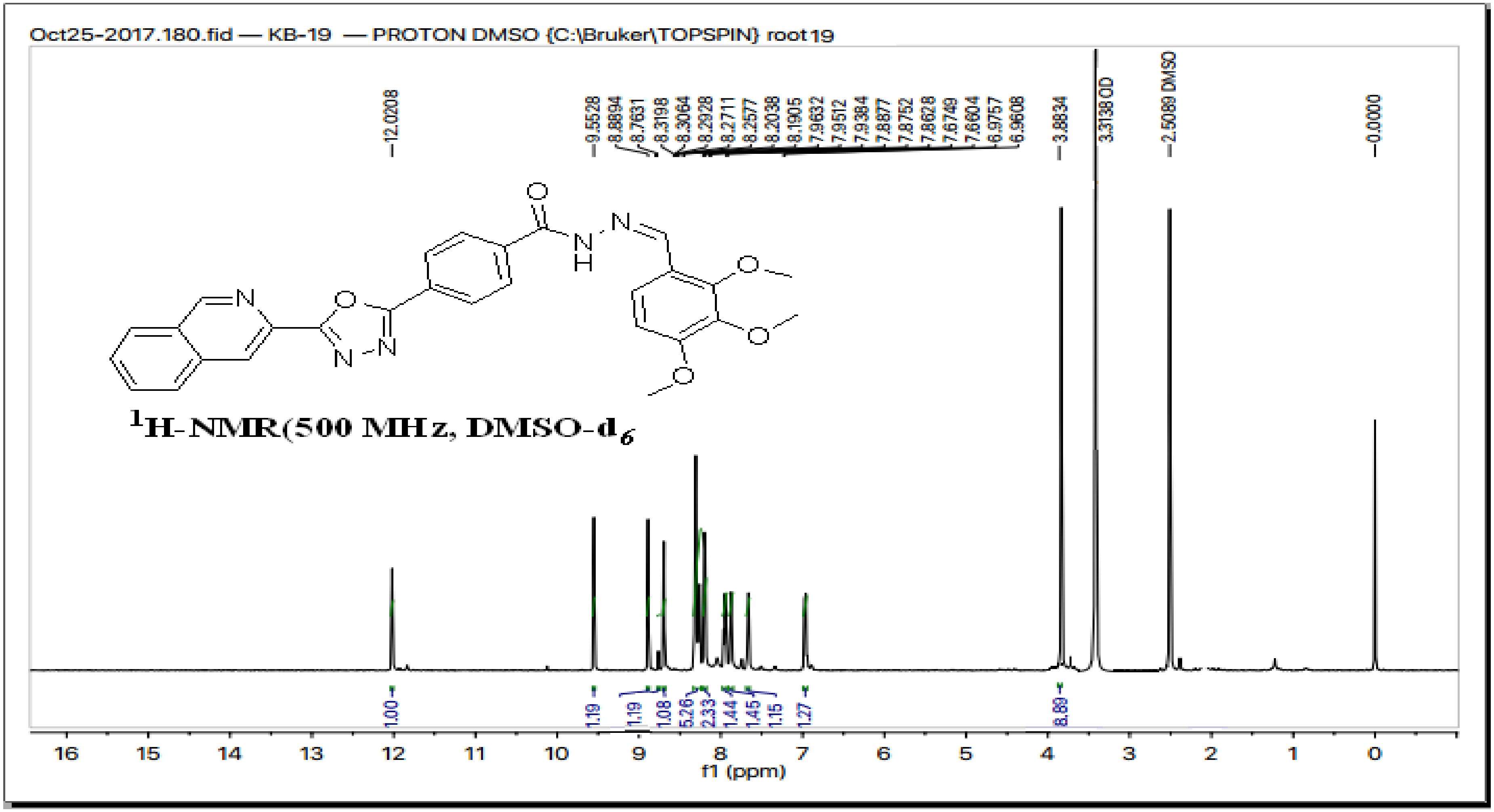

Supplementary Data

Compound NO-19

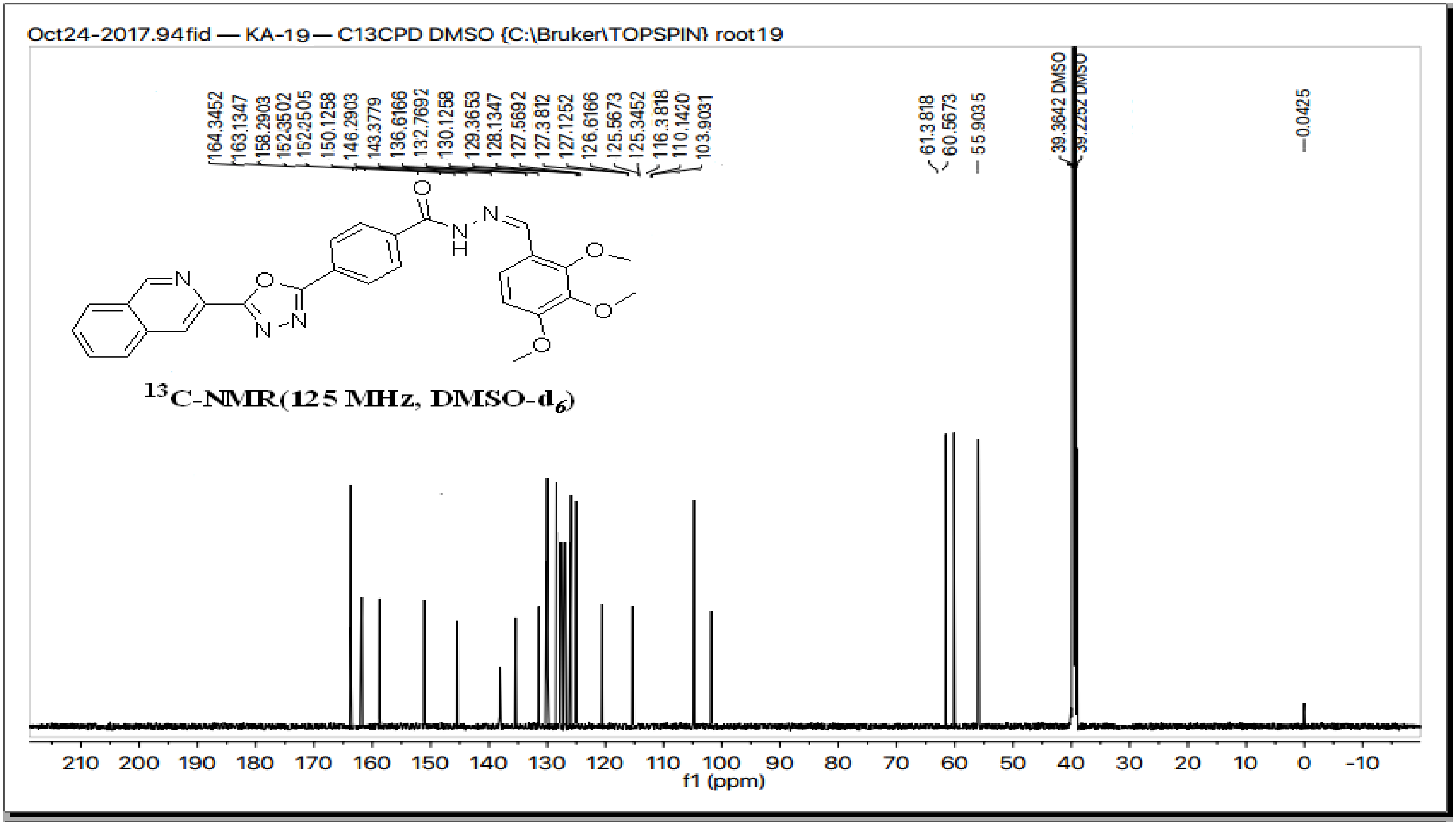

Supplementary Data

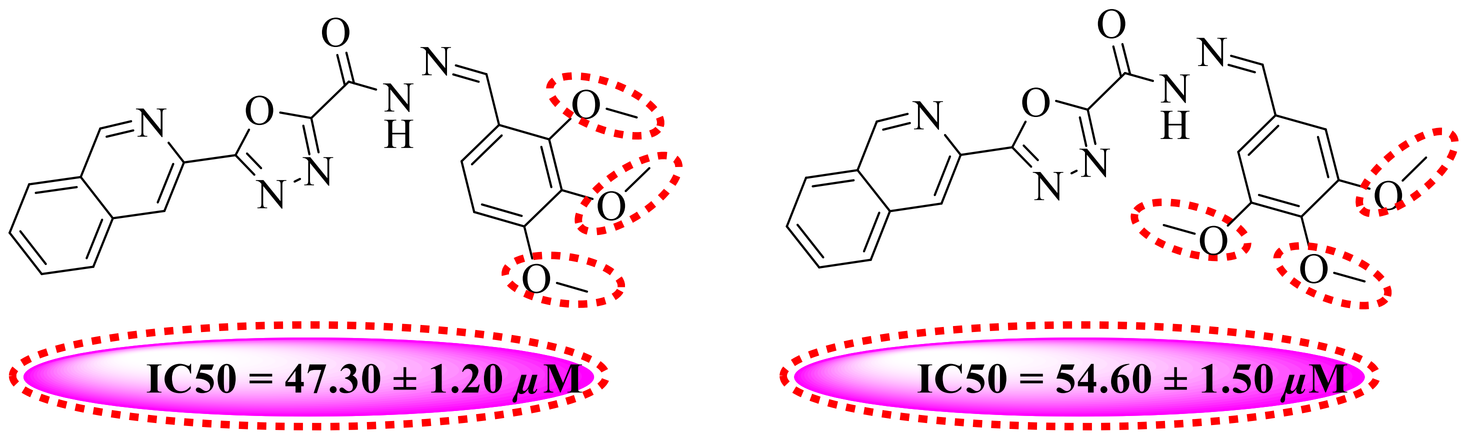

15

19

Figure S1: SAR of analog 15 and 19

Supplementary Data

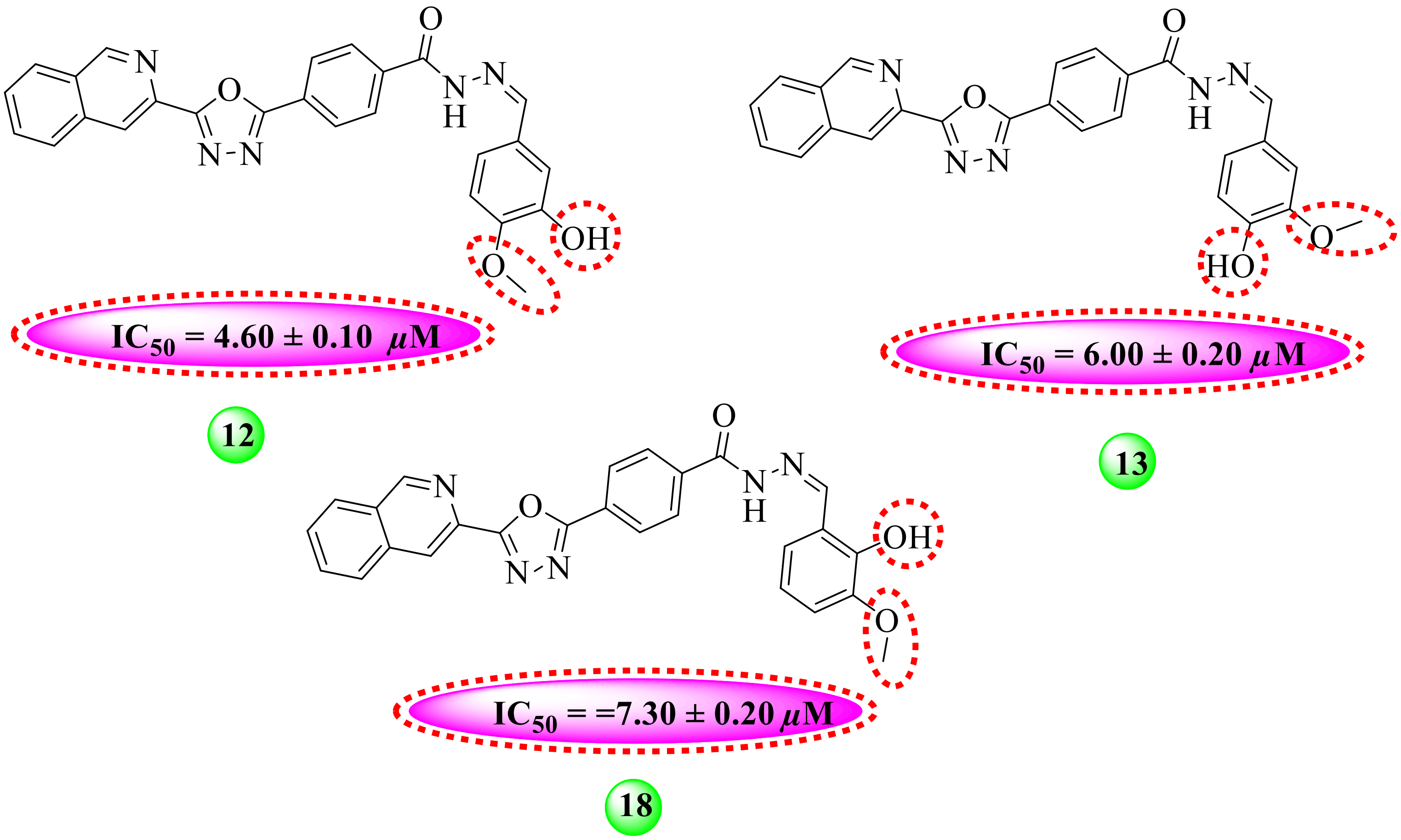

Figure-S2: SAR of analog 12, 13 and 18

Supplementary Data

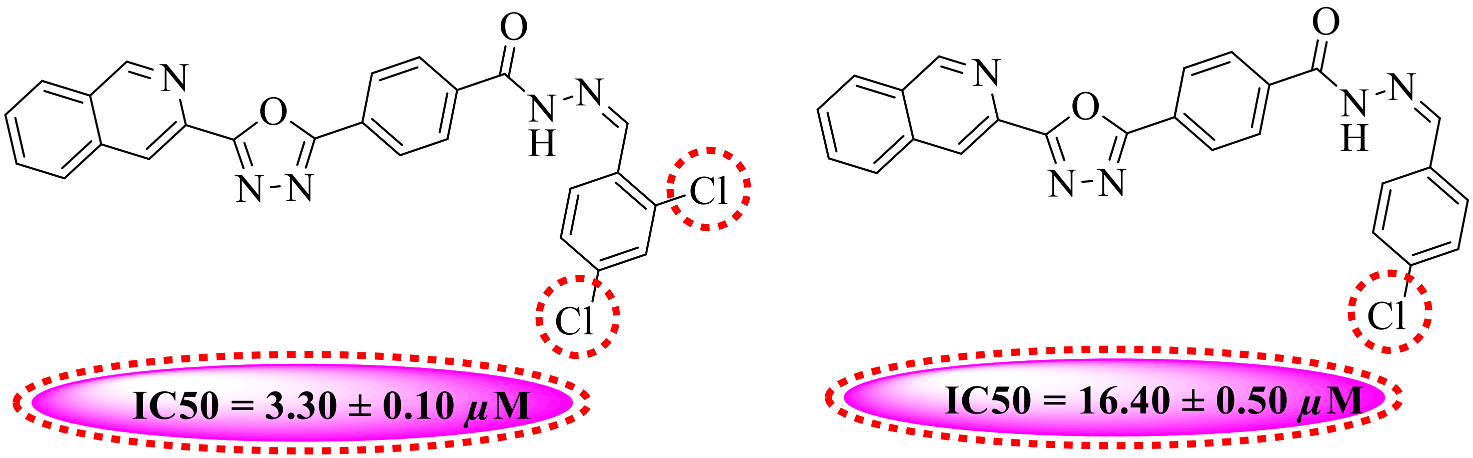

3 6

Figure-S3: SAR of analog 3 and 6

Supplementary Data

Table-S1: The detail report of docking score and dock conformation of predicted binding interactions

**Compouds**	**Docking score**	**Binding interaction report**				
	-11.7345	**Ligand**	**Receptor**	**Interaction**	**Distanc**	**E(kcal/**
		C 11	O GLY 121 (A)	H-donor	**e**	**mol)**
		C 30	O ARG 115(A)	H-donor	3.01	-0.7
		O 33	CD2 LEU117 (A)	H-acceptor	3.54	-0.3
		N 36	CB ALA 373 (A) 6-	H-accepter	3.92	-0.1
**1**		C 41	ring TYR168(A)	H-pi	2.92	-0.1
		6-ring	CB SER 86 (A)	pi-H	4.75	-0.1
		6-ring	N GLY 88 (A)	pi-H	4.51	-0.1
		6-ring	CA GLY 88 (A)	pi-H	4.29	-0.1

Supplementary Data

		6-ring	N GLY 114 (A)	pi-H	4.22	-0.5
		6-ring	N THR 123 (A)	pi-H	4.11	-0.6
					3.52	-0.1
**2**	-8.2349	C 1	OG SER 217 (A)	H-donor	2.61	-0.1
		C 3	O LYS 204(A)	H-donor	2.88	-0.4
		N 19	NH1 ARG 17 (A)	H-acceptor	3.63	-0.4
		O 33	NZ LYS 190 (A)	H-acceptor	3.15	-1.0
		6-ring	CE HIS 85 (A)	Pi-H	3.13	-0.1
		6-ring	6-ringTYR168 (A)	pi-pi	3.33	-0.0
**3**	-12.1135	C 1	O ILE 112 (A)	H-donor	3.59	-0.1
		C 3	SD MET 111 (A)	H-donor	3.43	-0.5
		C 5	OG SER 95 (A)	H-donor	2.95	-0.3
		C 11	OD2 ASP 92 (A)	H-donor	2.85	-0.3
		N 18	N GLY 89 (A)	H-accepter	3.22	-0.9
		O 33	OH TYR168 (A)	H-accepter	3.01	-0.2
		O 33	CG1VAL 177 (A)	H-accepter	3.64	-0.1
		CL 48	CB LEU 117 (A)	H-acceptor	3.52	-0.1
		CL 48	CD2 LEU 117 (A)	H-acceptor	3.92	-0.2
		C 28	6-ring PHE 210 (A)	H-pi	3.19	-0.1
		6-ring	CB SER 86 (A)	pi-H	4.08	-0.1
		6-ring	CB SER 86 (A)	pi-H	3.37	-0.3

Supplementary Data

		6-ring	CA GLY 122 (A)	pi-H	3.87	-0.1
		6-ring	N THR 123 (A)	pi-H	4.46	-1.5
		6-ring	N THR 123 (A)	pi- H	3.95	-1.5
**4**	-9.2210	N 18	CA ALA 373 (A)	H-accepter	3.65	-0.1
		N 19	CD1 LEU 117 (A)	H-accepter	3.64	-0.1
		O 33	CG1 VAL 177 (A)	H-accepter	3.81	-0.1
		N 36	CD1 ILE 183 (A)	H-accepter	4.14	-0.1
		6-ring	CG LYS 165 (A)	Pi-H	4.92	-0.1
		6-ring	CB ALA 373 (A)	Pi-H	4.28	-0.2
		6-ring	6-ring PHE 210 (A)	pi-pi	3.60	-0.0
		6-ring	6-ring TYR168 (A)	pi-pi	3.69	-0.0
**5**	-10.8892	C 5	OD2 ASP 83 (A)	H-donor	2.49	-0.4
		C 15	OG SER 95 (A)	H-donor	2.68	-0.1

		C 25	O ARG 115 (A)	H-donor	2.77	-0.1
		C 46	OD2 ASP 172(A)	H-donor	3.84	-0.1
		C 48	OH TYR 168(A)	H-donor	3.77	-0.1
		N 18	NZ LYS 84 (A)	H-accepter	3.96	-0.1
		O 33	CB LEU 117 (A)	H-accepter	3.00	-0.3
		O 54	CB ALA 373 (A)	H-accepter	3.07	-0.1
		6-ring	CB LYS 84 (A)	pi-H	3.95	-0.2
		6-ring	CD LYS 84 (A)	pi-H	3.97	-0.2
		6-ring	CD LYS 84 (A)	pi-H	4.04	-0.2
		5-ring	N GLY 88 (A)	pi-H	4.55	-0.1
		6-ring	CA SER 113 (A)	pi-H	4.27	-0.5
		6-ring	CD1 LEU 117 (A)	pi-H	4.64	-0.1
**6**	-10.6309	C 3	OG SER 95 (A)	H-donor	3.25	-0.1
		N 18	CG2 THR 120 (A)	H-accepter	3.88	-0.1
		N 19	CG2 THR 120 (A)	H-accepter	3.60	-0.2
		N 19	CD1 PHE 210 (A)	H-accepter	3.52	-0.2
		O 33	CB ASP 172 (A)	H-accepter	2.87	-0.1
		O 33	CG2 VAL 177 (A)	H-accepter	3.88	-0.1
		C 11	5-ring HIS 85 (A)	H-pi	4.68	-0.2
		6-ring	NZ LYS 84 (A)	pi-cation	4.73	-2.0
		6-ring	N GLY 88 (A)	pi-H	3.77	-0.2

		6-ring	N GLY 114 (A)	pi-H	4.46	-0.1
		6-ring	N THR 123 (A)	pi-H	3.82	-0.1
		6-ring	CE2 PHE 210 (A)	pi-H	3.17	-0.2
		6-ring	6-ring PHE 210 (A)	pi-pi	3.66	-0.0
**7**	-8.7652	C 30	OG1 THR 120 (A)	H-donor	3.17	-0.2
		C 41	SD MET 111 (A)	H-donor	2.65	-0.2
		C 43	O ILE 112 (A)	H-donor	2.99	-0.6
		6-ring	CA GLY 88 (A)	Pi-H	4.12	-0.1
		5-ring	CB LEU 117 (A)	Pi-H	4.47	-0.1
		5-ring	CD1 LEU 117 (A)	Pi-H	3.74	-0.1
		6-ring	CG1 VAL 177 (A)	Pi-H	4.70	-0.2
		6-ring	CG1 VAL 177 (A)	Pi-H	4.19	-0.2
**8**	-10.1273	C 3	O E1 GLU 194 (A)	H-donor	2.99	-0.1
		C 5	OE1 GLU 194 (A)	H-donor	2.97	-0.1
		C 25	O VAL 177 (A)	H-donor	3.27	-0.5
		N 34	OD2 ASP 172 (A)	H-donor	2.98	-2.1

Supplementary Data

		N 13	CA ILE 187 (A)	H-accepter	2.65	-0.1
		N 19	CA ILE 183 (A)	H-accepter	3.79	-0.1
		6-ring	OH TYR 168 (A)	pi-H	3.43	-0.1
		6-ring	5-ring HIS 85 (A)	pi-pi	3.82	-0.0
		6-ring	5-ring HIS 85 (A)	pi-pi	2.83	-0.0
**9**	-12.2195	C 28	OG1 THR 120 (A)	H-donor	3.55	-0.1
		C 37	O SER 86 (A)	H-donor	3.22	-0.2
		C 42	O VAL 177 (A)	H-donor	3.62	-0.1
		CL 47	CG1 VAL 177(A)	H-accepter	3.89	-0.2
		CL 47	CD2 PHE 210 (A)	H-accepter	3.70	-0.1
		CL 48	NH2 ARG171(A)	H-accepter	3.19	-0.8
		CL 48	CG1 ILE 183(A)	H-accepter	3.99	-0.2
		CL 48	CB SER 186 (A)	H-accepter	4.15	-0.1
		5-ring	CB LEU 117(A)	pi-H	4.30	-0.1
		6-ring	CB LEU 117(A)	pi-H	4.21	-0.1
		5-ring	CD1 LEU 117 (A)	pi-H	4.08	-0.4
		5-ring	CD2 LEU 117 (A)	pi-H	4.20	-0.4
		6-ring	CG2 ILE 183 (A)	pi-H	4.02	-0.4
		6-ring	CA GLN 372 (A)	pi-H	3.68	-0.3
		6-ring	CB ALA 373 (A)		4.23	-0.7

**10**	-13.7203	C 23	O LEU 117 (A)	H-donor	3.60	-0.1
		C 25	OG1 THR 120(A)	H-donor	3.52	-0.1
		C 30	OD2 ASP 172 (A)	H-donor	3.02	-0.1
		C 43	O VAL 177 (A)	H-donor	3.35	-0.1
		C 45	O VAL 177 (A)	H-donor	3.40	-0.1
		N 49	OD1 ASP 178 (A)	H-donor	1.85	-1.4
		N 49	SD MET 211 (A)	H-donor	3.39	-1.7
		N 36	CA THR 87 (A)	H-donor	2.59	-0.2
		5-ring	CB LEU 117 (A)	pi-H	3.87	-0.3
		5-ring	CD1 LEU 117 (A)	pi-H	4.35	-0.4
		5-ring	CD2 LEU 117(A)	pi-H	4.39	-0.1
		6-ring	CB ASP 172 (A)	pi-H	3.62	-0.2
		6-ring	CG1 ILE 183 (A)	pi-H	3.72	-0.2
		6-ring	CB ALA 373 (A)	pi-H	3.33	-0.3
**11**	-11.8934	C 1	OG SER 374 (A)	H-donor	3.14	-0.1
		C 55	OD1 ASP 172 (A)	H-donor	3.45	-0.1
		O 33	C PHE 210 (A)	H-accepter	3.26	-0.1
		6-ring	CG2 ILE 183 (A)	pi-H	4.76	-0.1
		6-ring	N ALA 373 (A)	pi-H	2.86	-0.1
		6-ring	6-ring PHE 210 (A)	pi-pi	3.56	-0.0
**12**	-12.0978	C 28	O ARG 115 (A)	H-donor	3.50	-0.4

Supplementary Data

		C 37	O SER 86 (A)	H-donor	3.03	-0.1
		O 33	CA GLY 88 (A)	H-acceptor	2.85	-0.2
		6-ring	NE2 HIS 85 (A)	pi-H	4.57	-0.2
		5-ring	CB LEU 117 (A)	pi-H	4.26	-0.7
		6-ring	CD2LEU 117 (A)	pi-H	4.99	-0.2
		6-ring	CG2 ILE 173 (A)	pi-H	3.72	-0.2
		6-ring	CD1 ILE 187 (A)	pi-H	4.18	-0.5
		6-ring	6-ring PHE 210 (A)	pi-pi	3.77	-0.0
**13**	-11.6789	C 1	O LYS 84 (A)	H-donor	2.76	-0.4
		C 3	SD MET 111 (A)	H-donor	3.25	-0.1
		C 30	OG1 THR 120 (A)	H-donor	2.93	-0.7
		C 40	OD2 ASP 172 (A)	H-donor	3.48	-0.1
		O 48	O ILE 183 (A)	H-donor	3.41	-0.1
		N 13	CA GLY 122 (A)	H-accepter	3.43	-0.4
		C 9	5-ring HIS 85 (A)	H-pi	3.69	-1.4
		6-ring	NZ LYS 84 (A)	pi-cation	4.69	-0.7
		6-ring	NZ LYS 84 (A)	pi-cation	3.91	-0.7
		5-ring	CA GLY 88 (A)	pi-H	3.67	-0.5
		6-ring	N GLY 114 (A)	pi-H	4.39	-0.1
		6-ring	N THR 123 (A)	pi-H	4.03	-0.1
		6-ring	NH2 ARG 171 (A)	pi-cation	3.48	-0.9

Supplementary Data

**14**	-9.4592	C 3	OG SER 95 (A)	H-donor	3.20	-0.2
		N 19	CB LEU 117 (A)	H-acceptor	3.64	-0.1
		N 19	CD1 LEU 117(A)	H-acceptor	4.04	-0.1
		O 33	OH TYR 168 (A)	H-acceptor	3.02	-0.7
		N 34	6-ring PHE 210(A)	H-pi	4.06	-0.1
		5-ring	CD1LEU 117 (A)	pi-H	4.75	-0.1
		6-ring	CAGLY 118 (A)	pi-H	4.83	-0.1
		5-ring	OG THR 120 (A)	pi-H	3.92	-0.1
		6-ring	N THR 123 (A)	pi-H	4.17	-0.1
		6-ring	N THR 123 (A)	pi-H	2.91	-0.1
**15**	-8.0012	C 25	O ARG 115 (A)	H-donor	3.66	-0.1
		C 58	SD MET 211 (A)	H-donor	4.37	-0.2
		N 13	CZ PHE 210 (A)	H-accepter	3.74	-0.1
		6-ring	CA THR 87 (A)	pi-H	4.39	-0.1
		6-ring	CA ALA 175 (A)	pi-H	3.51	-0.5
		6-ring	CA ALA 175 (A)	pi-H	4.70	-0.5
		6-ring	CD1 ILE 187 (A)	pi-H	3.88	-0.1
**16**	-10.0013	C 1	O ASP 92 (A)	H-donor	3.00	-0.1
		C 5	OG SER 95 (A)	H-donor	2.59	-0.1

		C	25	OGI THR 120 (A)	H-donor	2.76	-0.4
		N	13	N SER 86 (A)	H-accepter	3.09	-0.4
		6-ring		NZ LYS 84 (A)	Pi-cation	4.29	-0.5
		6-ring		OG SER 113 (A)	pi-H	3.16	-0.1
		6-ring		CG2 VAL 177 (A)	pi-H	4.35	-0.2
**17**	-9.8699	N	19	N LEU 117 (A)	H-accepter	3.40	-0.1
		N	19	CB LEU 117 (A)	H-accepter	3.82	-0.1
		N	36	CB ASP 172 (A)	H-accepter	3.68	-0.1
		N	36	CG1 VAL 177(A)	H-accepter	3.87	-0.1
		6-ring		CB SER 86 (A)	pi-H	4.81	-0.1
		6-ring		N GLY 114 (A)	pi-H	4.85	-0.1
		6-ring		N THR 123 (A)	pi-H	2.89	-0.1
		6-ring		N THR 123 (A)	pi-H	2.94	-0.1
		6-ring		CG1 VAL177(A)	pi-H	4.41	-0.1
**18**	-11.2703	C	37	O SER 86 (A)	H-donor	3.23	-0.2
		N	18	CG2 VAL177(A)	H-acceptor	3.72	-0.2
		N	19	CG2 VAL 177(A)	H-accepter	3.93	-0.1
		O	50	CE LYS 190(A)	H-accepter	3.21	-0.1
		C	51	6ring TYR168(A)	H-pi	3.42	-0.3
		5-ring		CB ASP 172 (A)	pi-H	3.81	-0.7
		6-ring		CG2 ILE 183 (A)	pi-H	4.88	-0.1

Supplementary Data

		6-ring	CD1 ILE 187 (A)	pi-H	4.34	-0.1
		6-ring	6-ring PHE210(A)	pi-pi	3.62	-0.0
**19**	-7.5431	N 13	CZ PHE 210 (A)	H-acceptor	4.07	-0.1
		C 48	5-ring HIS 85 (A)	H-pi	4.03	-1.5
		6-ring	CA GLY 114 (A)	pi-H	3.37	-0.1
		6-ring	CD2 LEU 117(A)	pi-H	3.89	-0.2
		6-ring	CB ASP 172 (A)	pi-H	4.56	-0.1
		5-ring	6-ringPHE 210(A)	pi-pi	3.89	-0.0
**20**	-13.9027	O 51	OG SER 186 (A)	H-donor	3.69	-2.2
		N 13	CDI LEU 117 (A)	H-accepter	3.22	-0.1
		OH 53	NH IYS 190 (A)	H-donor	1.91	-2.2
		N 36	CG LYS 165 (A)	H-accepter	2.13	-0.2
		O 52	O TYR 168 (A)	H-donor	3.01	-0.3
		NH	O TYR 168 (A)	H-donor	3.91	-0.3
		C	O ASP 172 (A)	H-accepter	3.8	-0.3
		6-ring	CD2 LEU117(A)	pi-H	5.05	-0.1
		6-ring	CA ALA 169 (A)	pi-H	3.90	-0.3
		6-ring	CA GLN 372 (A)	pi-H	3.58	-0.3
		6-ring	CB ALA 373 (A)	pi-H	3.98	-0.1
		6-ring	6ringTYR168(A)	pi-pi	3.55	-0.0
		6-ring	CDI PHE 210 (A)	H-donor	3.39	-1.7

Supplementary Data

**Standard7 -DX**	-8.2301	N 1	OG SER 186 (A)	H-donor	3.18	-2.3
		O 7	NZ LYS 190 (A)	H-accepter	2.61	-10.4
		O 8	NH1 ARG171(A)	H-accepter	3.28	-2.3
		O 8	NH2 ARG171(A)	H-accepter	3.16	-4.0
		6-ring	NE2 HIS 85 (A)	pi-H	4.64	-1.9
